# Factors associated with atrial functional tricuspid regurgitation severity: a comprehensive three-dimensional echocardiographic study

**DOI:** 10.1093/ehjimp/qyag088

**Published:** 2026-05-11

**Authors:** Kyu Kim, Seo-Yeon Gwak, Hyun-Jung Lee, Iksung Cho, Geu-Ru Hong, Jong-Won Ha, Chi Young Shim

**Affiliations:** Division of Cardiology, Severance Cardiovascular Hospital, Yonsei University College of Medicine, 50–1 Yonsei-ro, Seodaemun-gu, Seoul 03722, Republic of Korea; Division of Cardiology, Severance Cardiovascular Hospital, Yonsei University College of Medicine, 50–1 Yonsei-ro, Seodaemun-gu, Seoul 03722, Republic of Korea; Division of Cardiology, Severance Cardiovascular Hospital, Yonsei University College of Medicine, 50–1 Yonsei-ro, Seodaemun-gu, Seoul 03722, Republic of Korea; Division of Cardiology, Severance Cardiovascular Hospital, Yonsei University College of Medicine, 50–1 Yonsei-ro, Seodaemun-gu, Seoul 03722, Republic of Korea; Division of Cardiology, Severance Cardiovascular Hospital, Yonsei University College of Medicine, 50–1 Yonsei-ro, Seodaemun-gu, Seoul 03722, Republic of Korea; Division of Cardiology, Severance Cardiovascular Hospital, Yonsei University College of Medicine, 50–1 Yonsei-ro, Seodaemun-gu, Seoul 03722, Republic of Korea; Division of Cardiology, Severance Cardiovascular Hospital, Yonsei University College of Medicine, 50–1 Yonsei-ro, Seodaemun-gu, Seoul 03722, Republic of Korea

## Abstract

**Aims:**

Geometric alterations in the tricuspid annulus (TA) are common in atrial functional tricuspid regurgitation (A-FTR), and may reflect structural and dynamic changes that contribute to disease severity.

**Methods and results:**

Comprehensive two-dimensional and 3D echocardiographic analyses were performed in 216 patients with A-FTR. Geometric parameters included indexed TA area, sphericity index, and tenting volume. Dynamic parameters included TA area fraction, TA excursion, and unfavourable TA dynamics. A-FTR groups. Indexed TA area and tenting volume increased significantly with A-FTR severity (all *P* < 0.001). Severe A-FTR showed a higher prevalence of unfavourable TA dynamics (55.8%, *P* = 0.013) compared to mild (33.3%) and moderate (32.1%) groups. No significant differences were observed in TA area fraction and excursion across groups. In multivariable analysis, indexed TA area (aOR: 1.216, 95% CI: 1.166–1.267, *P* < 0.001), tenting volume (aOR: 1.150, 95% CI: 1.099–1.204, *P* < 0.001), and unfavourable TA dynamics (aOR: 1.309, 95% CI: 1.089–1.572, *P* = 0.005) were independently associated with A-FTR severity. TA geometry correlated with both RA (β=0.841, *P* < 0.001) and RV (β=0.748, *P* < 0.001) volumes, while unfavourable dynamics were associated only with RV volume (β=0.166, *P* = 0.027).

**Conclusion:**

3D remodelling and unfavourable TA dynamics independently correlated with A-FTR severity. TA geometry was linked to RA volume, while dynamics reflected RV remodelling. These findings support a potential sequential mechanism, highlighting the value of comprehensive 3D assessment.

## Graphical Abstract

**Graphical Abstract:**
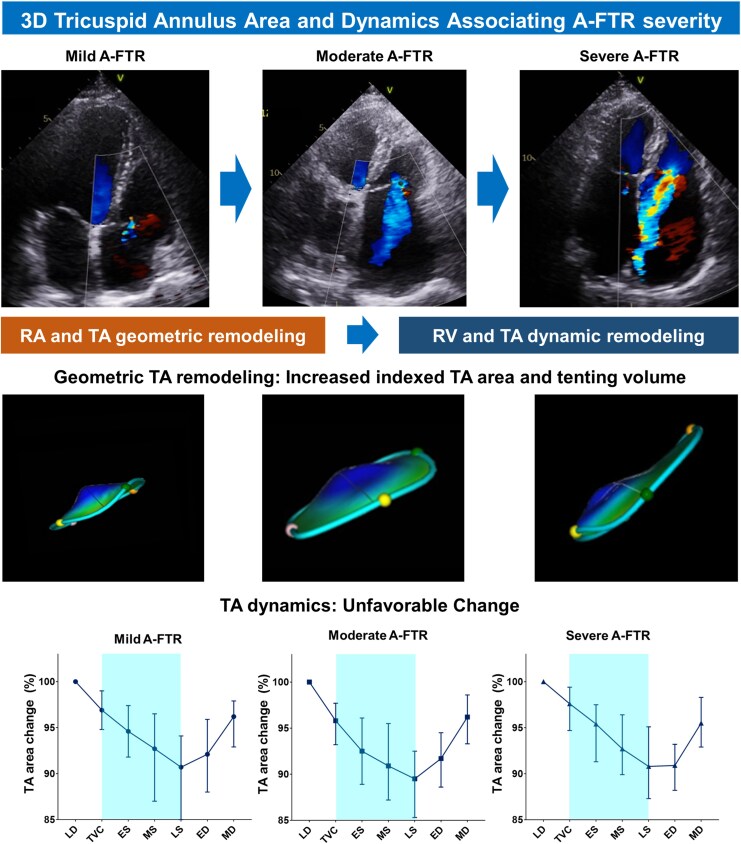
3D echocardiographic parameters associated with atrial functional tricuspid regurgitation severity.

## Introduction

Functional tricuspid regurgitation (FTR) caused by tricuspid annular (TA) dilatation and leaflet tethering is associated with poor clinical outcomes.^1–3^ Among various types of FTR, atrial FTR (A-FTR) is considered to have a distinct pathophysiology, typically occurring in the absence of primary pulmonary hypertension or significant left-sided valve disease.^4,5^ Atrial fibrillation (AF) is a key risk factor for A-FTR, and TA enlargement secondary to right atrial (RA) dilation has been proposed as the main mechanism in its development.^3,6^

Three-dimensional (3D) echocardiography is particularly important in assessing the severity of TR and the structure of the TA and right heart chambers, due to their complex, nonplanar, and asymmetric geometry.^7,8^ Notably, previous studies have shown that A-FTR is associated with a large, circular TA and less prominent leaflet tethering compared with ventricular FTR (V-FTR).^6,9^ However, most previous studies only focused on geometric remodelling of the TA. Although TA has been shown to exhibit dynamic features such as area fraction and longitudinal excursion, the relationship between TA dynamics and A-FTR severity remains poorly understood. Recent advances in 3D echocardiographic software have enabled analysis of both TA dynamics and geometry.

Therefore, we hypothesized that both TA geometry and dynamics are associated with A-FTR severity, and sought to explore their haemodynamic and structural correlates. We performed a comprehensive 3D echocardiographic assessment of TR severity, annular characteristics, and volumetric chamber parameters.

## Methods

### Study population

This retrospective cross-sectional study was conducted at a single tertiary centre. A total of 685 patients with long-standing AF and at least mild TR were retrospectively identified between July 2022 and February 2023. All patients had 3D echocardiographic datasets of sufficient quality for analysis. Long-standing AF was determined by reviewing electrocardiograms and medical records. Patients with the following conditions that could cause significant TR other than AF were excluded: previous heart intervention or surgery; concomitant significant left-sided valve disease (≥ moderate; *n* = 208); presence of a cardiac implantable device or primary tricuspid valve pathology (*n* = 134); left ventricular ejection fraction < 50% (*n* = 67); estimated pulmonary artery systolic pressure > 50 mmHg (*n* = 37); primary right ventricular (RV) disease (e.g. cardiomyopathy or infarction); and congenital heart disease (*n* = 4).^10^ In addition, patients with significant stitching artefacts that precluded 3D analysis were excluded (*n* = 19). Finally, a total of 216 A-FTR patients were analysed. This study was conducted in accordance with the Declaration of Helsinki, approved by the institutional review board, and the requirement for informed consent was waived due to the retrospective design.

### Echocardiographic assessments

All subjects underwent comprehensive two-dimensional (2D) and three-dimensional (3D) transthoracic echocardiography using Vivid E95 scanners (GE Vingmed Ultrasound, Horten, Norway), equipped with M5S and 4Vc probes. Standard 2D and Doppler measurements were performed in accordance with the American Society of Echocardiography guidelines.^11,12^ Pulmonary artery systolic pressure (PASP) was estimated by adding the Doppler-derived tricuspid regurgitation pressure gradient to the estimated RA pressure, which was assessed based on inferior vena cava diameter and respiratory variation. A full-volume, multi-beat 3D dataset of the RA, tricuspid valve (TV), and right ventricle (RV) was acquired using the indexed-beat method in patients with a heart rate below 100 beats per minute.^8,13^ The acquisition volume was adjusted to include the entire tricuspid apparatus and surrounding landmarks (e.g. septum, RV outflow tract, aortic valve). Gain, sector width, and depth were optimized to enhance temporal and spatial resolution. To ensure adequate image quality and temporal resolution (>15 volumes/sec, mean 21 ± 5), 3D non-colour datasets were acquired over four consecutive cardiac cycles. Colour Doppler datasets were acquired over seven consecutive cardiac cycles during breath-hold.^8,14^ Datasets were deemed feasible if they allowed accurate and reproducible analysis with clear visualization of the TA and minimal stitching artefacts. All 3D datasets were exported to a dedicated workstation and analysed offline using EchoPAC 206 software (GE Vingmed Ultrasound). All measurements were performed by a single experienced cardiologist (K.K.) without knowledge of the clinical data. RV volumes and ejection fraction were analysed using 4D Auto RVQ (see Supplementary data online, *Figure S1A*). RA volumes and emptying fraction were measured using 4D Auto LAQ (see Supplementary data online, *Figure S1B*), as there is no dedicated software for RA analysis. Tricuspid annular geometry and dynamics were assessed using 4D Auto TVQ (see Supplementary data online, *Figure S1C*).

Geometric parameters included the indexed TA area (TA area/body surface area), sphericity index (2CH TA diameter/4CH TA diameter × 100), and mid-systolic tenting volume. Dynamic parameters included TA area fraction {[(maximum − minimum TA area)/maximum TA area] × 100}, mid-systolic longitudinal excursion, and unfavourable TA dynamics. Unfavourable TA dynamics were defined as the minimum TA area occurring during diastole rather than systole. This pattern reflects discoordinated annular motion and blunted systolic annular contraction.^15^ The 4D Auto TVQ software provides frame-by-frame annular area measurements, enabling dynamic assessment across the cardiac cycle. TA area was measured at seven standardized time points: (1) early diastole (the frame after TV opening), (2) mid-diastole (the middle frame between early and late diastole), (3) late diastole (the frame before TV closure), (4) TV closure, (5) early systole (the frame after TV closed), (6) mid-systole (the middle frame between early and late systole), and (7) late systole (the frame before TV opening).^16^ Intra- and inter-observer reproducibility of TA area, sphericity index, tenting volume, area fraction, excursion, and unfavourable TA dynamics were assessed in 40 randomly selected patients. Inter-observer variability was evaluated by an independent observer (S.-Y.G.), and intra-observer variability was assessed by repeating measurements four weeks later by the same observer (K.K.).

TR severity was classified as mild, moderate, or severe according to the current guidelines by two experienced cardiologists (K.K. and C.Y.S.).^12,17^ An integrative approach incorporating qualitative, semiquantitative, and quantitative parameters was used. For semiquantitative assessment, the average vena contracta (VC) width was measured using the RV-inflow and the RV-focused apical view. For quantitative assessment, the proximal isovelocity surface area (PISA) method was used to calculate the effective regurgitant orifice area and regurgitant volume. However, in patients with mild TR, effective regurgitant orifice area and regurgitant volume were not reported because inadequate formation of the PISA shell often precluded reliable measurement. Accordingly, TR severity in these patients was assessed using vena contracta width rather than PISA-based quantitative parameters. In patients with adequate 3D colour Doppler imaging, 3D VC area (VCA) was also measured. A midsystolic frame with a measurable VCA was selected. Two orthogonal longitudinal planes were aligned parallel to the direction of the regurgitant jet, and the transverse plane was adjusted to the level of the smallest cross-sectional area.^17–19^ VCA was measured by direct planimetry of the aliased jet area in the transverse plane (see Supplementary data online, *Figure S1D*). Among the total study population, 37 patients (17.1%) had non-quantifiable 3D colour Doppler data and were therefore recorded as missing values.

### Statistical analysis

Data are reported as mean ± standard deviation (SD) or median (interquartile range) for continuous variables, depending on their distribution, and as number (percentage) for categorical variables. Normality was assessed using the Shapiro–Wilk test. Differences among groups were compared using the analysis of variance or the Kruskal–Wallis test, as appropriate. Post hoc analysis was performed using the Tukey or Bonferroni method, as appropriate. Receiver operating characteristic (ROC) analysis was performed to identify cutoff values associated with severe TR. The Cochran–Armitage trend test and the Jonckheere–Terpstra test were used to assess trends in binary and ordinal variables, respectively, according to TR severity. Univariable and multivariable linear regression analyses were performed to identify the factors independently associated with A-FTR severity. Tenting volume was analysed separately from tenting height and TA area due to collinearity. Multivariable model 1 included clinical variables along with indexed TA area, tenting height, sphericity index, and dynamic TA parameters. Multivariable model 2 included tenting volume instead of indexed TA area and tenting height. Linear or logistic regression was used to assess associations between conventional echocardiographic parameters and 3D TA geometric and dynamic indices. To statistically compare standardized beta coefficients obtained from separate regression models with the same dependent variable, a Z-test for independent regression coefficients was performed. Intraclass correlation coefficients (ICCs) were calculated to assess the inter-observer and intra-observer variability of the 3D TA parameters (indexed TA area, TA area fraction, sphericity index, and TA excursion). Since unfavourable TA dynamics is a binary variable, both inter- and intra-observer variability were assessed using observed agreement and Cohen’s kappa coefficient. All tests were two-sided, and a *P*-value < 0.05 was considered statistically significant. All statistical analyses were conducted using R version 4.1.0 (The R Foundation for Statistical Computing, www.R-project.org).

## Results

### Baseline characteristics

The mean age was 74.3 ± 9.8 years, and 95 subjects (44.0%) were female. Among the 216 patients with A-FTR, 111 (51.4%) had mild TR, 53 (24.5%) had moderate TR, and 52 (24.1%) had severe TR. *Table 1* summarizes the baseline clinical and echocardiographic characteristics according to TR severity. With increasing TR severity, both age and the proportion of females increased significantly (*P* < 0.001). The moderate and severe TR groups had significantly larger left atrial volumes than the mild group, whereas left ventricular ejection fraction and E/e’ did not differ significantly across groups. Estimated PASP also increased with TR severity (28.0 [26.0–35.0], 39.0 [35.0–46.0], and 42.0 [38.0–48.0] mmHg, respectively; *P* < 0.001). Regarding 3D echocardiographic measurements, RV volume indices were significantly larger in the severe TR group compared to the other groups, but no significant difference was observed between the mild and moderate groups. RA maximum and minimum volume indices progressively increased with TR severity (*P* < 0.001). Compared with the mild group, RA emptying fraction and RA/RV end-systolic volume ratio were significantly lower in the moderate and severe TR groups compared to the mild group, but did not differ significantly between the two. The VC area and VC width increased significantly across TR severity categories.

**Table 1:** Baseline clinical and echocardiographic characteristics of study populations

	Mild TR (*n* = 111)	Moderate TR (*n* = 53)	Severe TR (*n* = 52)	*P*-value
***Clinical characteristics***				
Age, years	70.5 ± 10.2	77.9 ± 6.8*	78.8 ± 8.0*	<0.001
Female sex, *n* (%)	32 (28.8)	31 (58.5)*	32 (61.5)*	<0.001
Body surface area, m^2^	1.76 ± 0.19	1.61 ± 0.16*	1.60 ± 0.17*	<0.001
Hypertension, *n* (%)	83 (74.8)	42 (79.2)	30 (57.7)^*†^	0.030
Diabetes mellitus, *n* (%)	27 (24.3)	8 (15.1)	16 (30.8)	0.162
Chronic kidney disease, *n* (%)	13 (11.7)	9 (17.0)	8 (15.4)	0.618
Coronary artery disease, *n* (%)	10 (9.0)	6 (11.3)	1 (1.9)	0.165
Heart rate, bpm	73.5 [63.5–84.0]	73.0 [65.0–82.0]	70.0 [64.0–78.0]	0.437
***2D and Doppler echocardiographic characteristics***				
LV ejection fraction, %	63.0 [58.0–68.0]	63.0 [57.0–68.0]	64.5 [59.0–69.5]	0.447
LA volume index, mL/m^2^	52.5 [43.8–64.1]	74.4 [59.4–93.1]*	76.5 [61.0–97.2]*	<0.001
E/e`	12.0 [9.0–15.0]	13.0 [11.0–15.0]	13.0 [10.0–15.5]	0.100
Estimated PASP, mmHg	28.0 [26.0–35.0]	39.0 [35.0–46.0]*	42.0 [38.0–48.0] ^*†^	<0.001
TR Vmax, m/s	2.4 ± 0.2	2.7 ± 0.4	2.6 ± 0.3	0.050
Effective orifice area, cm^2‡^	NA	0.34 [0.21–0.38]	0.77 [0.37–1.31]	<0.001
Regurgitant volume, ml^‡^	NA	21.5 [17.7–28.1]	55.6 [29.4–87.2]	<0.001
Average VC width, mm	2.1 [1.0–3.1]	6.0 [4.8–6.5]*	11.7 [8.8–14.5]^*†^	<0.001
***3D echocardiographic characteristics***				
3D RV end-diastolic volume index, mL/m^2^	58.5 [50.4–69.6]	63.7 [53.8–79.1]	100.6 [73.9–127.8]^*†^	<0.001
3D RV end-systolic volume index, mL/m^2^	30.6 [25.0–39.4]	30.2 [25.1–39.5]	51.5 [36.6–67.9]^*†^	<0.001
3D RV Ejection fraction, %	47.0 [44.0–52.5]	52.0 [46.5–55.0]*	51.0 [44.0–55.0]	<0.001
3D RA maximum volume index, mL/m^2^	38.2 [28.8–50.5]	58.2 [43.2–71.6]*	98.2 [78.7–140.1]^*†^	<0.001
3D RA minimum volume index, mL/m^2^	29.3 [22.3–37.6]	43.3 [34.1–59.1]*	79.1 [60.8–117.2]^*†^	<0.001
3D RA emptying fraction, %	22.0 [18.0–28.0]	19.5 [15.0–24.0]*	19.0 [16.0–23.0]*	0.009
3D RA/RV end-systolic volume ratio	1.2 [1.0–1.6]	1.7 [1.4–2.4]*	1.9 [1.5–2.4]*	<0.001
3D VC area, mm^2^	13.0 [3.0–22.0]	45.0 [35.0–59.0]*	126.0 [94.0–198.0]^*†^	<0.001
3D Indexed TA area, cm^2^/m^2^	5.3 [4.6–6.1]	7.3 [6.5–7.8]*	9.0 [7.6–11.3]^*†^	<0.001
Sphericity index, %	79 [72–86]	80 [75–87]	84 [80–90]*	0.006
3D Tenting volume, ml	1.8 [1.5–2.4]	2.2 [1.7–3.1]*	3.8 [2.7–6.0]^*†^	<0.001
3D Tenting height, mm	6.0 [6.0–8.0]	7.0 [5.0–8.0]	8.0 [7.0–1.1]^*†^	<0.001
3D TA area fraction, %	13.5 [9.0–17.9]	13.3 [9.4–16.3]	11.5 [9.0–14.5]*	<0.001
TA excursion, mm	5.0 [4.0–7.0]	5.0 [4.0–7.0]	6.0 [4.0–8.0]	0.537
Unfavourable TA dynamics, *n* (%)	37 (33.3)	17 (32.1)	29 (55.8)^*†^	0.013

2D, two-dimensional; LV, left ventricle; LA, left atrium; E/e’, the ratio of early diastolic mitral inflow velocity to early diastolic mitral annular velocity; PASP, pulmonary artery systolic pressure, 3D, three-dimensional; RV, right ventricle; RA, right atrium; VC, vena contracta; TA, tricuspid annulus; *P < 0.05 compared with the group of mild TR; †*P* < 0.05 compared with the group of moderate TR; ‡There were missing values in 91 (42.1%) of study population.

### 3D TA geometry and dynamics

**Figure 1:**
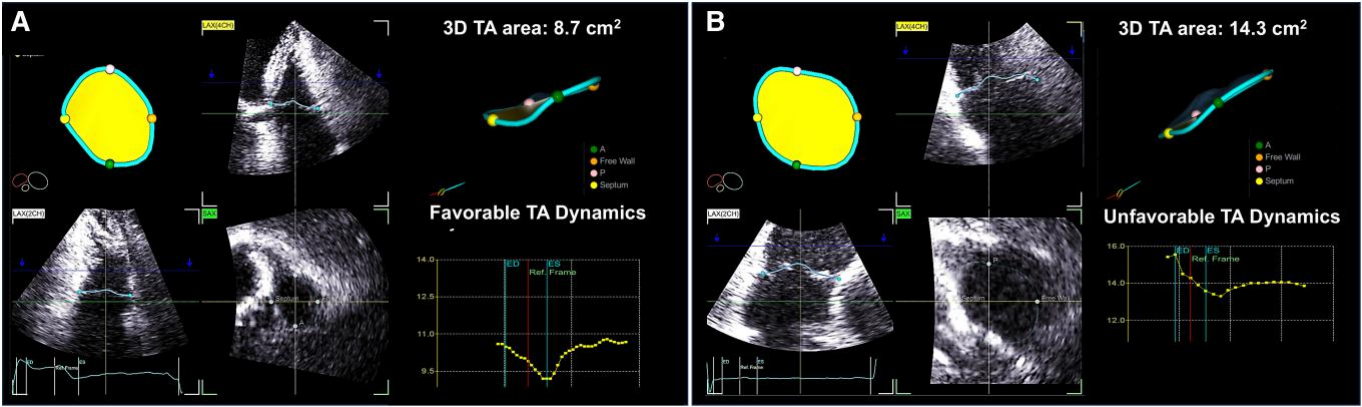
Measurement of 3D geometric (left panel) and dynamic parameters (right panel) of TA in mild A-FTR (*A*) and severe A-FTR (*B*). A-FTR, atrial functional tricuspid regurgitation. *X-axis of right lower graph: time, *Y*-axis of right lower graph: TA area.

*Figure 1* illustrates typical examples of TA geometry and dynamics in patients with mild and severe A-FTR. Patients with mild A-FTR exhibited a smaller 3D TA annulus, lower tenting volume, and favourable TA dynamics (*Figure 1A*), whereas those with severe A-FTR showed a markedly enlarged annulus, increased tenting volume, and unfavourable dynamics (*Figure 1B*). There were no significant differences in baseline or echocardiographic characteristics between the unfavourable and favourable TA dynamics subgroups within the mild TR group (see Supplementary data online, *Table S1*).

**Figure 2:**
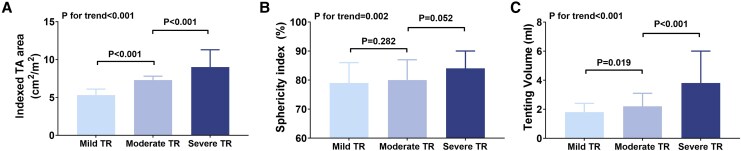
The geometric TA parameters based on the A-FTR severity. (*A*) Indexed TA area, (*B*) sphericity index, (*C*) tenting volume.

*Figure 2* presents the distribution of 3D TA geometric parameters across TR severity groups. The median indexed TA area was 5.3 (4.6–6.1) cm^2^/m^2^ in mild TR, 7.3 (6.5–7.8) cm^2^/m^2^ in moderate TR, and 9.0 (7.6–11.3) cm^2^/m^2^ in severe TR (*P* for trend < 0.001). The median sphericity index was 79% (72–86%) in mild TR, 80% (75–87%) in moderate TR, and 84% (80–90%) in severe TR (*P* for trend = 0.002). There was no significant difference between the mild and moderate TR groups. The sphericity index was greater in severe TR than in moderate TR, although the difference did not reach statistical significance (*P* = 0.052). Tenting volume increased significantly with TR severity (*P* for trend < 0.001). ROC analysis identified cutoff values associated with severe TR of 8 mm for tenting height, 6.5 cm^2^/m^2^ for indexed TA area, and 2.6 mL for tenting volume

**Figure 3:**
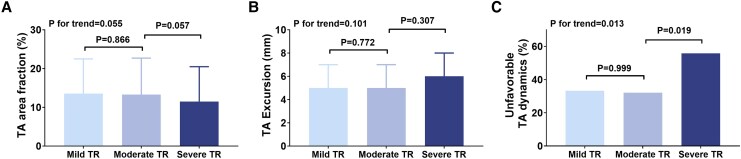
The dynamic TA parameters based on the A-FTR severity. (*A*) TA area fraction, (*B*) TA excursion, (*C*) unfavourable TA dynamics.

*Figure 3* displays TA dynamic parameters across TR severity groups. There were no significant differences in TA area fraction (*P* for trend = 0.055) or excursion (*P* for trend = 0.101) across TR severity groups. However, the TA area fraction tended to be lower in severe TR than in moderate TR (*P* = 0.057). Unfavourable TA dynamics were significantly more prevalent in the severe TR group (55.8%) compared with the mild (33.3%) and moderate (32.1%) groups (*P* for trend = 0.013).

**Figure 4:**
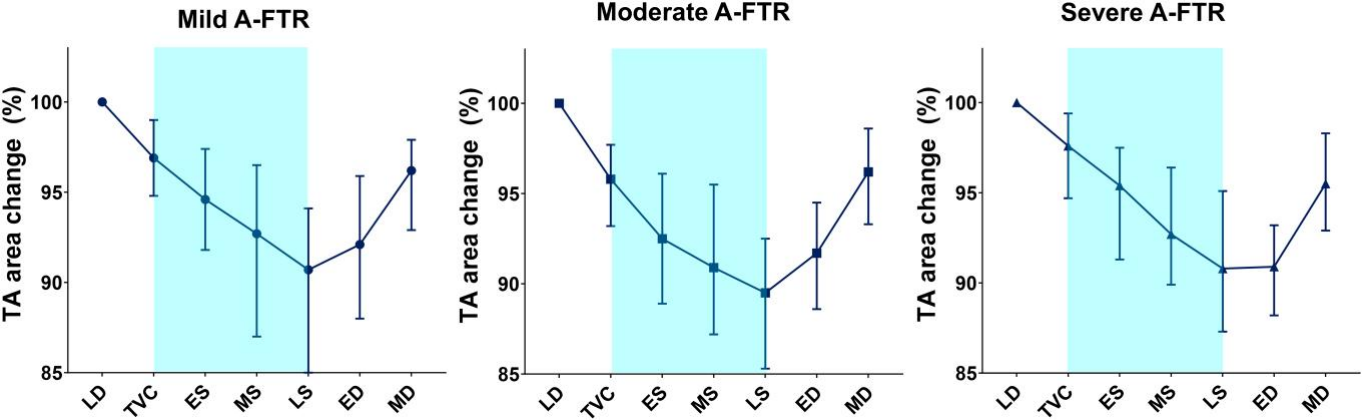
Changes in tricuspid annular area during the cardiac cycle according to A-FTR severity. Abbreviations: TA, tricuspid annulus; A-FTR, atrial functional tricuspid regurgitation; LD, late diastole; TVC, tricuspid valve closure; ES, early systole; MS, mid-systole; LS, late systole; ED, early diastole; MD, mid-diastole.

*Figure 4* shows temporal changes in TA area across cardiac phase. The minimum TA area most commonly occurred in late systole in the mild and moderate TR groups (46.8% and 45.3%, respectively), but shifted to early diastole in the severe TR group (51.9%). All 3D TA parameters showed excellent inter-observer and intra-observer agreement (see Supplementary data online, *Figure S2*). The interobserver and intraobserver agreements for unfavourable TA dynamics were 92.5% (κ = 0.846) and 95.0% (κ = 0.898), respectively.

### Factors associated with severe TR and adverse 3D TA parameters

The clinical and 3D echocardiographic factors associated with A-FTR severity are shown in *Table 2*. Among geometric parameters, indexed TA area [adjusted odds ratio (aOR): 1.216, 95% CI: 1.166–1.267, *P* < 0.001] and tenting volume (aOR: 1.150, 95% CI: 1.099–1.204, *P* < 0.001) were independently associated with TR severity after multivariable adjustment. Tenting height and sphericity index were not significantly associated with TR severity. In TA dynamic parameters, neither the TA area fraction nor excursion was significantly associated with TR severity. However, unfavourable TA dynamics was independently associated with TR severity (aOR: 1.309, 95% CI: 1.089–1.572, *P* = 0.005). Between the mild and moderate TR groups, geometric parameters such as indexed TA area and tenting volume were significantly associated with TR severity, whereas functional parameters, including TA area fraction, annular excursion, and unfavourable dynamics were not (see Supplementary data online, *Table S2*). In contrast, between the moderate and severe TR groups, both unfavourable dynamics and geometric parameters were significantly associated with TR severity (see Supplementary data online, *Table S3*).

**Table 2:** The associated clinical and 3D echocardiographic factors with a-FTR severity

	Univariable		Multivariable model 1		Multivariable model 2	
	OR (95% CI)	*P* value	Adjusted OR (95% CI)	*P* value	Adjusted OR (95% CI)	*P* value
***Clinical variables***						
Age, years	1.033 (1.022–1.044)	<0.001	1.017 (1.008–1.027)	<0.001	1.022 (1.011–1.032)	<0.001
Female sex	1.628 (1.316–2.015)	<0.001	1.463 (1.209–1.770)	<0.001	1.417 (1.142–1.759)	0.002
Body surface area, m^2^	0.261 (0.150–0.455)	<0.001	1.449 (0.820–2.561)	0.203	0.693 (0.373–1.290)	0.249
***3D geometric parameters***						
Indexed TA area, cm^2^/m^2^	1.258 (1.218–1.300)	<0.001	1.216 (1.166–1.267)	<0.001	—	—
Tenting height, mm	2.759 (1.866–4.079)	<0.001	0.994 (0.707–1.399)	0.974	—	—
TA Sphericity index, %	1.017 (1.006–1.028)	0.002	1.005 (0.997–1.013)	0.415	1.008 (0.999–1.017)	0.067
Tenting volume	1.206 (1.153–1.260)	<0.001	—	—	1.150 (1.099–1.204)	<0.001
***3D dynamic parameters***						
TA area fraction, %	0.978 (0.960–0.996)	0.019	0.997 (0.984–1.011)	0.696	0.989 (0.975–1.005)	0.174
TA excursion, mm	1.435 (0.921–2.236)	0.112	1.291 (0.916–1.820)	0.146	1.374 (0.931–2.028)	0.114
Unfavourable TA dynamics	1.333 (1.065–1.667)	0.013	1.270 (1.081–1.493)	0.004	1.309 (1.089–1.572)	0.005

3D, three-dimensional; TA, tricuspid annulus

*Table 3* summarizes the associations between conventional echocardiographic variables and adverse 3D TA parameters. The 3D indexed TA area was significantly associated with RV and RA volumes, RA emptying fraction, the RA/RV end-systolic volume ratio, and the LA volume index (all *P* < 0.05). The β coefficient of RA volume was higher than that of RV volume in its association with indexed TA area (Z = 27.3, *P* < 0.001). Tenting volume was significantly associated with RV and RA volumes, LA volume index, and estimated PASP (all *P* < 0.05). In this model, the β coefficient for RV volume was significantly greater than that for RA volume (Z = 15.0, *P* < 0.001). Unfavourable TA dynamics was associated with RV end-diastolic volume index (β = 0.177, *P* = 0.028), but not with RA volume index or emptying fraction. The β coefficients for RV volumes were significantly greater than those for RA volumes (Z = 59.7, *P* < 0.001). TA area fraction was significantly associated with RV ejection fraction (β = 0.262, *P* < 0.001), but not with RA emptying fraction (β = 0.123, *P* = 0.104). These results suggest that A-FTR severity is primarily influenced by bi-atrial enlargement and functional deterioration due to chronic AF. RV pressure and volume overload, as reflected by increased PASP and RV volumes, may contribute to further geometric remodelling of the TA.

**Table 3:** The associated conventional echocardiographic factors with 3D indexed TA area and unfavourable TA dynamics

	Indexed TA area		Tenting volume		Unfavourable TA dynamics	
	β coefficients	*P* value	β coefficients	*P* value	β coefficients	*P* value
RV end-diastolic volume index, mL/m^2^	0.723	<0.001	0.786	<0.001	0.177	0.018
RV end-systolic volume index, mL/m^2^	0.696	<0.001	0.771	<0.001	0.152	0.043
RV ejection fraction, %	0.094	0.217	0.039	0.607	0.055	0.465
RA maximum volume index, mL/m^2^	0.841	<0.001	0.724	<0.001	0.087	0.248
RA minimum volume index, mL/m^2^	0.828	<0.001	0.707	<0.001	0.073	0.332
RA emptying fraction, %	−0.179	0.018	−0.114	0.131	0.052	0.489
RA/RV end-systolic volume ratio	0.394	<0.001	0.129	0.088	−0.096	0.204
LV ejection fraction, %	−0.014	0.859	−0.039	0.602	−0.018	0.815
LA volume index, mL/m^2^	0.473	<0.001	0.193	0.010	−0.095	0.260
E/e`	−0.009	0.907	−0.093	0.216	−0.018	0.068
Estimated PASP, mmHg	0.500	<0.001	0.338	<0.001	0.004	0.963

3D, three-dimensional; PASP, pulmonary artery systolic pressure; RV, right ventricle; RA, right atrium; LV, left ventricle; LA, left atrium; E/e’, the ratio of early diastolic mitral inflow velocity to early diastolic mitral annular velocity

## Discussion

The present study demonstrated that 3D indexed TA area, tenting volume, and unfavourable TA dynamics were independently associated with A-FTR severity. Notably, the indexed TA area correlated more strongly with RA remodelling, whereas unfavourable dynamics were linked exclusively to RV changes. These findings suggest distinct but interrelated remodelling patterns in A-FTR, from atrial-driven geometric remodelling to RV-related annular dysfunction. These results, derived from a large dataset of 3D TA analyses, offer new insights into the respective roles of RA and RV remodelling in severe A-FTR. However, they should be interpreted in light of several important limitations, including the single-centre design, the ethnically homogeneous study population, and the TR quantification strategy based primarily on 3D VCA and 2D VC width.

### 3D TA geometry and the severity of a-FTR

FTR is the most common type of TV dysfunction, and TA dilation plays a central role in the development of TR.^20^ AF-related TR is typically accompanied by significant TA and RA enlargement without concurrent RV dilation.^20,21^ Consistent with these findings, our study demonstrated a progressive increase in indexed TA area and a trend towards increased sphericity index across TR severity.^21,22^ Muraru et al. showed that RA volume was the major determinant of TA area, with A-FTR patients exhibiting disproportionately greater RA enlargement compared to RV enlargement.^22^ These findings highlight that RA remodelling plays a predominant role in annular dilation.

Although RV size did not differ significantly between mild and moderate TR groups in our cohort, a marked increase was noted between the moderate and severe groups. TA geometric parameters correlated more strongly with RA than with RV, although significant associations with RV parameters were also observed. While prior studies have emphasized RA-driven mechanisms, our findings suggest that RV remodelling may play a more important role in advanced stages of A-FTR. In multivariable analysis, both indexed TA area and tenting volume were independently associated with A-FTR severity, whereas tenting height was not. Although A-FTR is primarily characterized by annular enlargement and flattening, increased tenting in severe TR may reflect superimposed RV remodelling. As RV dilatation progresses, leaflet tethering may increase because of outward displacement of the subvalvular apparatus, resulting in greater tenting height and volume despite the underlying atrial phenotype. This suggests that in the setting of established RA remodelling, even modest RV changes—insufficient to affect tenting height—may still increase tenting volume and contribute to disease progression.^5,23^ These findings underscore the importance of determining appropriate timing for intervention, ideally before significant RV changes occur. Early detection of annular geometric and dynamic alterations may support more timely and effective treatment strategies.

In addition to RA and RV remodelling, haemodynamic factors such as estimated PASP and LA volume may further influence TA geometry. Although estimated PASP was significantly associated with TA geometry, this relationship should be interpreted with caution, as PASP may reflect secondary effects of atrial and ventricular remodelling rather than being a primary driver. LA enlargement, a marker of AF duration,^24^ and elevated PASP often indicate underlying diastolic dysfunction and chronic haemodynamic burden.^25^ As A-FTR typically occurs in the absence of marked pulmonary hypertension, its pathophysiology likely involves combined right and left heart remodelling, reinforcing the need for early AF control and diastolic dysfunction prevention.

### 3D TA dynamics and the severity of A-FTR

Investigations into annular dynamics have been limited compared to geometric changes, which have been extensively studied. The advent of 3D echocardiographic software has enabled assessment of annular motion throughout the cardiac cycle, offering valuable insights into annular function.^26^ In the present study, dynamic parameters such as TA area fraction and unfavourable TA dynamics did not differ significantly between mild and moderate A-FTR but showed marked differences between moderate and severe TR groups. Both parameters were also significantly associated with RV volume, suggesting a potential role of RV remodelling in annular dysfunction. Prior studies support these observations. For instance, in patients with hypoplastic left heart syndrome, a reduced TA area fraction correlated with TR severity, largely attributed to RV remodelling and loss of ventricular interaction.^27^ Similarly, reduced mitral annular systolic area change has been linked to more severe MR and poorer LV function in patients with A-FMR.^16^ These findings suggest that RV remodelling may become a critical determinant of annular dynamics in later stages of A-FTR, particularly in the context of preexisting annular dilation.

The concept of ‘unfavourable TA dynamics’ refers to blunted annular contraction, with the minimum TA area occurring during diastole rather than mid- or end-systole as observed in normal physiology.^15,16,26^ Physiologically, unfavourable TA dynamics indicate that the TA fails to achieve its smallest area during systole, when leaflet coaptation is expected to be maximal. As a result, a relatively larger systolic annular size may reduce leaflet coaptation and contribute to increasing TR severity.^15^ This finding supports the concept that altered temporal annular mechanics, beyond static annular dilatation alone, may play an important role in the mechanism and progression of TR. Prior studies have shown similar temporal shifts in mitral annular dynamics, particularly in patients with AF or A-FMR.^16^ In the present study, unfavourable TA dynamics were observed in approximately 30% of patients with mild or moderate TR, likely due to the presence of AF in all patients. In a recent study in which TA dynamics in 35 AF patients and 35 subjects with sinus rhythm were compared, unfavourable TA dynamics were observed more frequently in AF and significant TR.^15^ The present study extends these findings by reproducing the association between unfavourable TA dynamics and A-FTR severity in a larger and ethnically diverse cohort of 216 patients. Moreover, a study by Deferm et al. demonstrated that restoration of sinus rhythm improved annular dynamics and reduced the severity of A-FMR, suggesting a possible mechanism by which rhythm control might help limit A-FTR progression.^28^ Furthermore, our results revealed a significant association between unfavourable TA dynamics and RV volume, whereas no such association was observed with RA volume, suggesting that ventricular remodelling plays a predominant role in annular function in advanced A-FTR. While RA enlargement is well recognized in the early stages of the disease,^5,21^ these findings indicate that RV changes may increasingly govern TA function as A-FTR progresses. Although V-FTR patients were excluded from the present study, it remains uncertain whether unfavourable TA dynamics are unique to A-FTR. However, given that previous studies have predominantly linked unfavourable dynamics to the presence of AF,^15,28^ these findings are likely to reflect a pathophysiologic mechanism specific to A-FTR.

## Clinical implications

In functional TR, interventions are often performed at an advanced stage, marked by right heart failure and older age, resulting in suboptimal outcomes and a high rate of residual TR.^29,30^ In this context, our findings provide additional mechanistic insight into the remodelling patterns associated with A-FTR severity. Previous 3D echocardiographic studies have suggested that atrial TR is characterized by relatively limited leaflet tethering and annular enlargement, with proposed cutoffs such as tenting height <9 mm, and tenting volume <2.5 mL.^9^ Notably, these values largely corresponded to the moderate TR group in our cohort. ROC analysis in our study identified tenting height of 8 mm, indexed TA area of 6.5 cm^2^/m^2^, and tenting volume of 2.6 mL as cutoff values associated with severe TR. These findings extend prior phenotypic descriptions by providing cohort-specific thresholds linked to more advanced disease expression. Our findings suggest that, in A-FTR, RA-associated annular enlargement and RV-associated annular dysfunction may reflect distinct but interrelated remodelling patterns. Patients with more severe TR showed greater evidence of RV remodelling and unfavourable annular dynamics. However, given the cross-sectional design of this study, these observations should be interpreted as hypothesis-generating rather than as proof of temporal progression or causality. The observed association between unfavourable TA dynamics and RV volume highlights the potential value of dynamic annular assessment for identifying patients with a more advanced remodelling phenotype before irreversible changes develop. In our study, the key difference between the moderate and severe TR groups was the presence of unfavourable annular dynamics, which were more closely associated with RV remodelling than with RA enlargement. This suggests that static structural parameters and dynamic annular function may provide complementary information for staging A-FTR. These insights may support earlier intervention strategies focused on annular function in the era of transcatheter tricuspid therapies. From a clinical perspective, this raises the possibility that patients with A-FTR may benefit from earlier intervention, including tricuspid edge-to-edge repair and rhythm control strategies, In the current era of transcatheter tricuspid therapies, these insights raise the possibility that patients with A-FTR may benefit from earlier intervention, including tricuspid edge-to-edge repair and rhythm control strategies, before irreversible ventricular remodelling and advanced annular dysfunction develop. Further prospective studies are warranted to determine the prognostic value of dynamic TA parameters and guide optimal treatment timing.

## Limitations

This study has several limitations. First, the cross-sectional design of this study precludes causal inference regarding the relationship between TR severity and 3D echocardiographic TA parameters. Therefore, the observed associations should be considered hypothesis-generating. Second, this was a single-centre study conducted at a tertiary referral hospital in South Korea, which may have introduced referral bias and limited the generalizability of the findings to other ethnic populations. Third, multi-beat 3D image acquisition was not feasible in some patients with high R-R variability. To minimize this limitation, only datasets with adequate temporal resolution and minimal stitching artefacts were included for analysis. Fourth, the use of vendor-specific software may limit generalizability, although the platform employed offers advanced and reproducible TA measurements. Fourth, the use of vendor-specific software may limit generalizability, although the platform employed offers advanced and reproducible TA measurements. Fifth, TR quantification was based on 3D VCA and average 2D VC width rather than effective regurgitant orifice area or regurgitant volume, owing to the known limitations of the PISA method in A-FTR. Although acquisition of 7-beat 3D colour Doppler datasets in patients with AF is technically challenging, beat-to-beat variability is often relatively limited in patients with long-standing AF, and 3D VCA measurement at the narrow regurgitant orifice level was frequently feasible despite minor stitching artefacts. Nevertheless, 3D colour Doppler data were non-quantifiable in 37 patients (17.1%) because of suboptimal image quality, mostly in the mild TR group, which may have affected measurement quality in some cases. In addition, AF-related factors such as AF burden, adequacy of rate control, and rhythm management strategies may have influenced tricuspid annular remodelling and TR severity. These variables were not systematically analysed in the present study, and therefore residual confounding cannot be excluded. Sixth, while the overall sample size was large, the distribution across TR severity groups was uneven, potentially impacting statistical comparisons. Seventh, RA 3D volume was assessed using LAQ software. Because this software has not been validated for RA analysis, there may have been limitations in measurement accuracy. Finally, all 3D measurements were based on the indexed beat, which may not fully account for beat-to-beat variability; nonetheless, long-standing AF patients typically exhibit relatively stable ventricular rates, supporting the reliability of this approach.^6^

## Conclusion

In this comprehensive 3D echocardiographic study, TA geometric remodelling and unfavourable annular dynamics were independently associated with A-FTR severity. TA geometry was more strongly associated with RA than with RV volume, whereas unfavourable dynamics were selectively associated with RV remodelling. These findings support a potential sequential progression in A-FTR, in which dynamic annular dysfunction may emerge alongside RV remodelling, following earlier geometric changes. Comprehensive 3D assessment of TA morphology and function may enhance mechanistic understanding and inform clinical decision-making in patients with A-FTR.

## Supplementary Material

qyag088_Supplementary_Data

## Data Availability

Data would be available upon reasonable request.

## Lead author biography

Professor Chi Young Shim is a Professor of Cardiology at Yonsei University College of Medicine. Her clinical and research interests focus on valvular heart disease and heart failure, with particular emphasis on echocardiography and multimodality imaging. She received her PhD from Yonsei University and is actively engaged in clinical research aimed at improving diagnosis, risk stratification, and management of patients with cardiovascular disease.
