# Supplementary material for: Factors associated with atrial functional tricuspid regurgitation severity: a comprehensive three-dimensional echocardiographic study
Source: Eur Heart J Imaging Methods Pract. 2026 May 11;4(1):qyag088. doi: 10.1093/ehjimp/qyag088 (PMC13239420; doi:10.1093/ehjimp/qyag088)
Supplement: qyag088_Supplementary_Data [file qyag088_supplementary_data.docx]

**Supplementary Figure 1.** Measurement of 3D echocardiographic dataset using dedicated software (EchoPAC 206, GE). (A) Quantitative analysis of RV geometry and function using 4D Auto RVQ. (B) Quantitative analysis of RA geometry and function using 4D Auto LAQ. (C) Quantitative analysis of TA geometry and function using 4D Auto TVQ. (D) Quantitative analysis of TR severity based on 3D VC area.
3D, three-dimensional; RA, right atrium; RV, right ventricle; TA, tricuspid annulus; VC, vena contracta


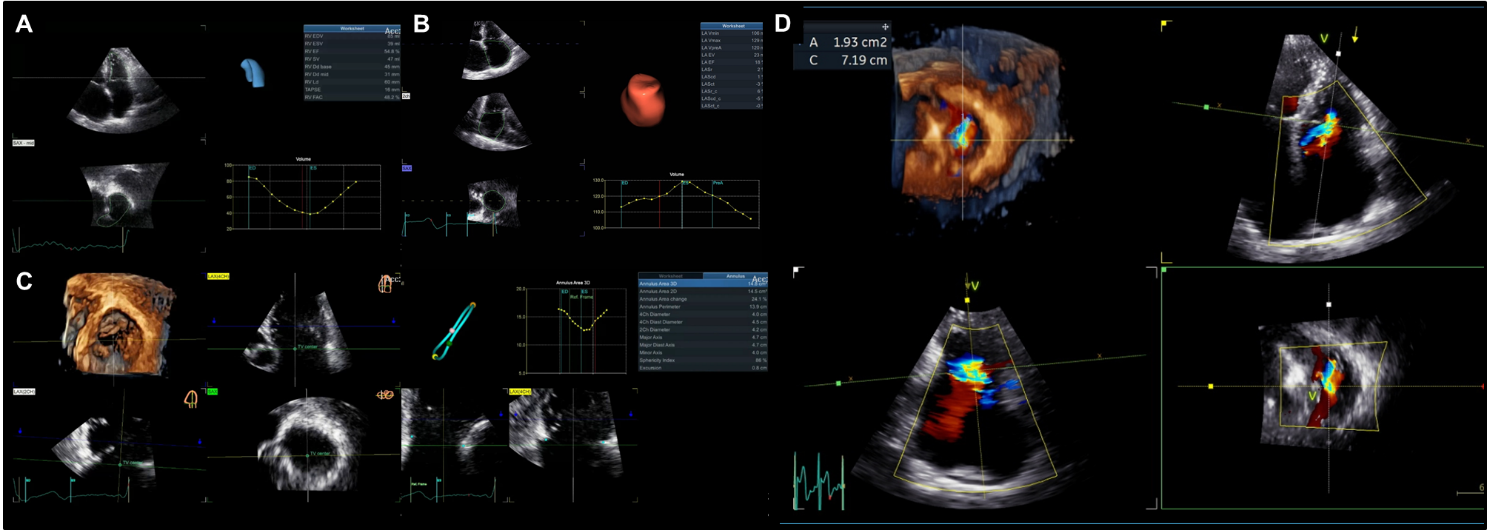


**Supplementary Figure 2.** Bland-Altman plot of inter-observer and intra-observer variability of 3D echocardiographic measurements.


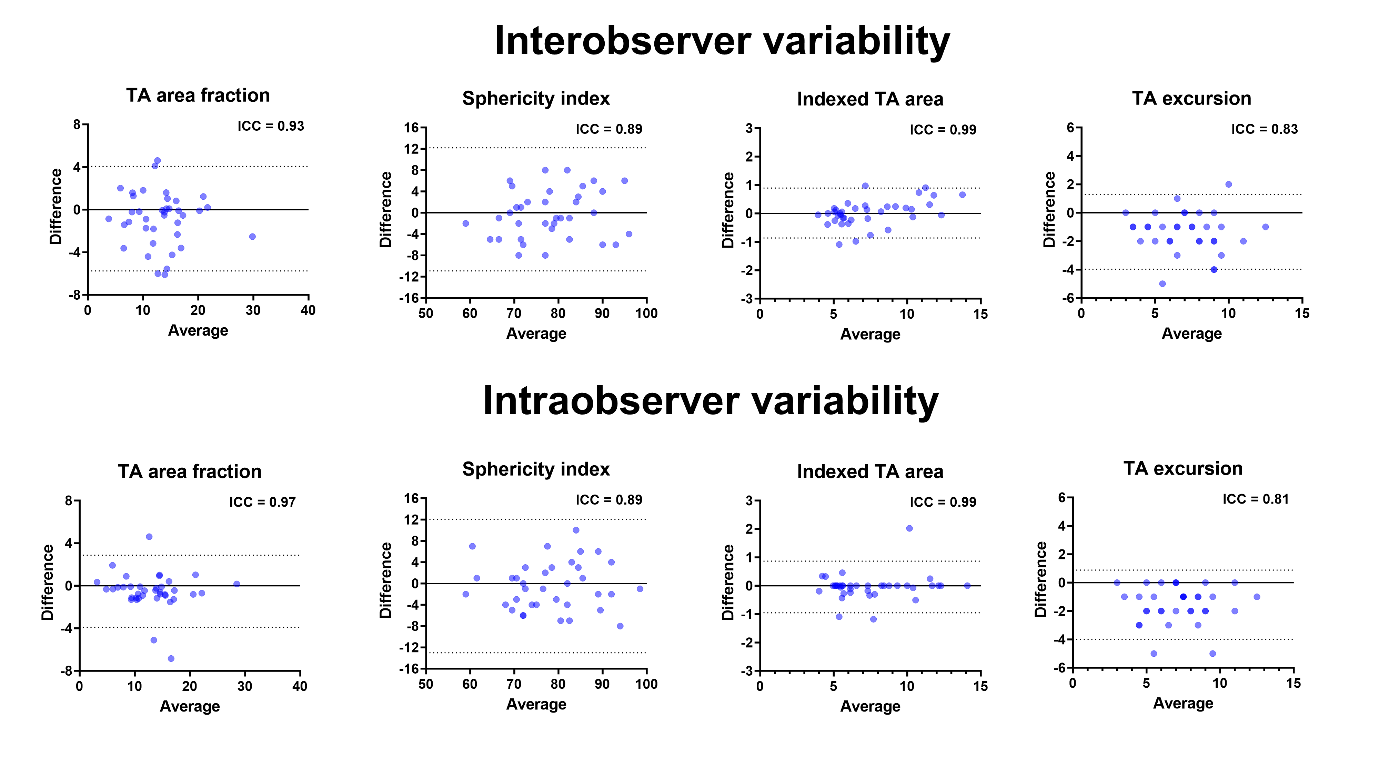


**Supplementary Table 1. The associated clinical and 3D echocardiographic factors with A-FTR severity. (mild vs. moderate)**

|  | **Univariable** | | **Multivariable** | | **Multivariable** | |
| --- | --- | --- | --- | --- | --- | --- |
|  | OR (95% CI) | P value | Adjusted OR  (95% CI) | P value | Adjusted OR  (95% CI) | P value |
| ***Clinical variables*** |  |  |  |  |  |  |
| Age, years | 1.017 (1.010-1.024) | <0.001 | 1.009 (1.002-1.017) | 0.014 | 1.012 (1.004-1.019) | 0.004 |
| Female sex | 1.316 (1.141-1.516) | 0.002 | 1.173 (1.009-1.363) | 0.039 | 1.133 (0.966-1.330) | 0.128 |
| Body surface area, m^2^ | 0.412 (0.292-0.583) | <0.001 | 1.063 (0.675-1.674) | 0.792 | 0.598 (0.383-0.936) | 0.026 |
| ***3D geometric parameters*** |  |  |  |  |  |  |
| Indexed TA area, cm^2^/m^2^ | 1.178 (1.132-1.227) | <0.001 | 1.160 (1.106-1.217) | <0.001 | - | - |
| Tenting height, mm | 0.989 (0.682-1.433) | 0.953 | 1.090 (0.785-1.513) | 0.607 | - | - |
| TA Sphericity index, % | 1.003 (0.996-1.010) | 0.355 | 1.001 (0.994-1.007) | 0.872 | 1.002 (0.996-1.009) | 0.529 |
| Tenting volume | 1.112 (1.027-1.203) | 0.009 | - | - | 1.163 (1.175-1.257) | <0.001 |
| ***3D dynamic parameters*** |  |  |  |  |  |  |
| TA area fraction, % | 0.998 (0.987-1.009) | 0.731 | 1.004 (0.994-1.015) | 0.393 | 1.002 (0.991-1.013) | 0.756 |
| TA excursion, mm | 0.885 (0.652-1.202) | 0.435 | 0.967 (0.729-1.282) | 0.813 | 0.985 (0.727-1.334) | 0.921 |
| Unfavorable TA dynamics | 0.963 (0.834-1.113) | 0.612 | 1.073 (0.946-1.217) | 0.275 | 1.041 (0.911-1.189) | 0.553 |

3D, three-dimensional; TA, tricuspid annulus

**Supplementary Table 2. The associated clinical and 3D echocardiographic factors with A-FTR severity. (moderate vs. severe)**

|  | **Univariable** | | **Multivariable** | | **Multivariable** | |
| --- | --- | --- | --- | --- | --- | --- |
|  | OR (95% CI) | P value | Adjusted OR  (95% CI) | P value | Adjusted OR  (95% CI) | P value |
| ***Clinical variables*** |  |  |  |  |  |  |
| Age, years | 1.004 (0.991-1.017) | 0.560 | 1.006 (0.995-1.018) | 0.276 | 1.005 (0.993-1.017) | 0.412 |
| Female sex | 1.032 (0.848-1.257) | 0.753 | 1.244 (1.015-1.525) | 0.038 | 1.195 (0.968-1.475) | 0.101 |
| Body surface area, m^2^ | 1.298 (0.718-2.345) | 0.389 | 1.536 (0.824-2.863) | 0.180 | 1.236 (0.653-2.341) | 0.517 |
| ***3D geometric parameters*** |  |  |  |  |  |  |
| Indexed TA area, cm^2^/m^2^ | 1.104 (1.067-1.142) | <0.001 | 1.078 (1.034-1.125) | <0.001 | - | - |
| Tenting height, mm | 1.790 (1.353-2.370) | 0.001 | 1.094 (0.795-1.506) | 0.582 | - | - |
| TA Sphericity index, % | 1.011 (1.001-1.021) | 0.039 | 1.003 (0.994-1.012) | 0.497 | 1.005 (0.996-1.015) | 0.283 |
| Tenting volume | 1.081 (1.048-1.115) | <0.001 | - | - | 1.056 (1.019-1.094) | 0.003 |
| ***3D dynamic parameters*** |  |  |  |  |  |  |
| TA area fraction, % | 0.979 (0.961-0.998) | 0.031 | 0.988 (0.972-1.005) | 0.158 | 0.985 (0.969-1.002) | 0.097 |
| TA excursion, mm | 1.493 (1.041-2.140) | 0.032 | 1.179 (0.841-1.653) | 0.342 | 1.121 (0.850-1.728) | 0.290 |
| Unfavorable TA dynamics | 1.462 (1.214-1.761) | <0.001 | 1.304 (1.096-1.550) | 0.004 | 1.348 (1.126-1.613) | 0.002 |

3D, three-dimensional; TA, tricuspid annulus

**Supplementary Table 3. The associated clinical and 3D echocardiographic factors with A-FTR severity. (mild+moderate vs. severe)**

|  | **Univariable** | | **Multivariable** | | **Multivariable** | |
| --- | --- | --- | --- | --- | --- | --- |
|  | OR (95% CI) | P value | Adjusted OR  (95% CI) | P value | Adjusted OR  (95% CI) | P value |
| ***Clinical variables*** |  |  |  |  |  |  |
| Age, years | 1.011 (1.006-1.017) | <0.001 | 1.006 (1.000-1.011) | 0.033 | 1.007 (1.001-1.013) | 0.016 |
| Female sex | 1.187 (1.060-1.329) | 0.033 | 1.192 (1.069-1.329) | 0.002 | 1.175 (1.046-1.321) | 0.007 |
| Body surface area, m^2^ | 0.682 (0.507-0.919) | 0.013 | 1.389 (1.004-1.920) | 0.048 | 0.991 (0.710-1.384) | 0.958 |
| ***3D geometric parameters*** |  |  |  |  |  |  |
| Indexed TA area, cm^2^/m^2^ | 1.115 (1.095-1.135) | <0.001 | 1.092 (1.066-1.118) | <0.001 | - | - |
| Tenting height, mm | 1.837 (1.506-2.240) | <0.001 | 1.139 (0.938-1.383) | 0.191 | - | - |
| TA Sphericity index, % | 1.009 (1.003-1.014) | 0.034 | 1.002 (0.997-1.006) | 0.455 | 1.004 (0.999-1.009) | 0.103 |
| Tenting volume | 1.109 (1.084-1.134) | <0.001 | - | - | 1.083 (1.057-1.109) | <0.001 |
| ***3D dynamic parameters*** |  |  |  |  |  |  |
| TA area fraction, % | 0.988 (0.979-0.997) | 0.013 | 0.997 (0.990-1.005) | 0.497 | 0.994 (0.986-1.002) | 0.154 |
| TA excursion, mm | 1.304 (1.037-1.639) | 0.024 | 1.140 (0.940-1.384) | 0.186 | 1.170 (0.950-1.440) | 0.141 |
| Unfavorable TA dynamics | 1.274 (1.140-1.423) | <0.001 | 1.185 (1.085-1.295) | 0.002 | 1.207 (1.097-1.327) | <0.001 |

3D, three-dimensional; TA, tricuspid annulus
